# Treatment Approaches to Eating Disorders Among LGBTQIA+ Population: A Narrative Review

**DOI:** 10.1192/j.eurpsy.2023.918

**Published:** 2023-07-19

**Authors:** S. Tempia Valenta, C. Bronte, F. Panariello, F. Bonazzoli, D. De Ronchi, A. R. Atti

**Affiliations:** Department of Biomedical and Neuromotor Sciences, University of Bologna, Bologna, Italy

## Abstract

**Introduction:**

Historically, eating disorders (ED) have been regarded as the diseases of heterosexual, affluent white women. Instead, research shows that the population most at risk of ED is lesbian, gay, bisexual, transgender, queer/questioning, intersex, and asexual/aromantic/agender (LGBTQIA+). Indeed, in addition to many of the same sociocultural influences on body dissatisfaction faced by their peers, LGBTQIA+ individuals experience unique body- and gender-related concerns as well as high levels of stress due to interpersonal prejudice and discrimination.

**Objectives:**

This narrative review presents an overview of current research on treatment approaches to ED among LGBTQIA+ individuals.

**Methods:**

We conducted a PubMed search for studies published after 1990 using terms that aimed to represent the primary concepts of “eating disorder” and “LGBTQIA+” and “therapy.” Next, we inductively created relevant macro-themes by synthesizing the data from the included articles.

**Results:**

Of 123 PubMed studies, we included 12 studies and identified three relevant macro-themes. The first macro-theme, “ordinary treatments,” focused on efficacy studies of conventional ED therapies applied to this category of patients. In particular, the first study proved the efficacy of the dissonance-based intervention, engaging participants to induce cognitive dissonance concerning the thin-ideal standard of beauty; the second study showed that sexual minorities patients accessing day hospital treatment reported greater overall ED and comorbid symptoms but started treatment with higher scores and improved at a faster rate compared to heterosexual patients; the third study provided evidence that transgender/nonbinary individuals and cisgender individuals showed similar improvement in ED symptoms during higher levels of care treatment, but the first group had less improvement in depression and no improvement in suicidality during ED treatment. The second macro-theme, “relational approach,” investigated newer treatment paradigms involving family and school support, both revealing positive implications for eating and weight-related behaviors. The third macro-theme, “gender-affirming therapy,” focused on medical and surgical treatment toward gender transition, which has been shown to correlate with improvements in body image, ED psychopathology, and psychological functioning.

**Conclusions:**

Members of the LGBTQIA+ community are at greater risk for ED; to our knowledge, there is no targeted treatment that considers the entirety of their experience. These findings denote the need to focus future research efforts on effective treatment strategies specific to sexual and gender identity groups.

**Disclosure of Interest:**

None Declared

